# Puberty status influences bacterial communities of the boar urogenital tract

**DOI:** 10.1093/jas/skaf336

**Published:** 2025-09-27

**Authors:** Brooke E McAnally, Dallas R Soffa, Molly S Smith, Kyle J Hickman, Olivia J Ognibene, Jeffrey G Wiegert, Rebecca K Poole

**Affiliations:** Department of Animal Science, Texas A&M University, College Station, TX 77843; Department of Animal Science, Texas A&M University, College Station, TX 77843; Department of Animal Science, Texas A&M University, College Station, TX 77843; Department of Animal Science, Texas A&M University, College Station, TX 77843; Department of Animal Science, Texas A&M University, College Station, TX 77843; Department of Animal Science, Texas A&M University, College Station, TX 77843; Department of Animal Science, Texas A&M University, College Station, TX 77843

**Keywords:** boar, microbiota, puberty, testosterone, urogenital tract

## Abstract

Bacteria present in fresh extended boar semen may impair the fertility of artificial insemination doses in swine. To date, information regarding the presence or composition of bacterial communities within the boar’s urogenital tract is lacking. These unexplored communities may contribute to the bacterial composition of semen and thereby influence boar fertility. Moreover, hormonal and anatomical changes that occur during puberty could also alter the urogenital tract bacterial communities. Therefore, the objective of this study was to evaluate pre- and post-pubertal shifts in bacterial communities and diversity in boar urogenital tissues (i.e., testis, epididymis, seminal vesicle, prostate, bulbourethral gland, bladder, and preputial diverticulum) using 16S rRNA gene amplicon community sequencing. Crossbred boars were euthanized at 74 ± 2 days of age (pre-pubertal; *n* = 4) or 276 ± 3 days of age (post-pubertal; *n* = 6), and intact reproductive tracts were harvested. Sterile swab samples were collected from each tissue of interest for microbiota analysis, and plasma was collected to analyze circulating hormone concentrations of testosterone and dihydrotestosterone. Circulating testosterone was greater (*P *< 0.01) in post-pubertal boars compared to pre-pubertal boars (3.01 ± 0.26 vs. 0.96 ± 0.36 ng/mL) yet no differences were observed in dihydrotestosterone concentrations. The relative abundance of the phylum Firmicutes was elevated (*P *< 0.05) in the testis, epididymis, seminal vesicle, bulbourethral gland, and preputial diverticulum of pre-pubertal boars and negatively correlated (*P *< 0.05) with testosterone. Alternatively, the relative abundance of the phylum Proteobacteria was greater in those same tissues from post-pubertal boars (*P *< 0.05) and was positively correlated (*P *< 0.05) with testosterone. Alpha-diversity was reduced in the urogenital tracts of post-pubertal boars compared to pre-pubertal boars (*P *< 0.01). The bladder had greater alpha-diversity compared to other tissues (*P *< 0.05). Pre- and post-pubertal boar urogenital tissues have distinct bacterial communities and shifts in these communities following puberty attainment may be associated with elevated testosterone. Future research is warranted to compare bacterial compositions of the boar urogenital tissues to the animal’s ejaculate, which would provide greater insight into the origin of bacteria within boar semen.

## Teaser

This research demonstrates that hormonal changes during puberty markedly reshape the boar urogenital microbiome. These insights pave the way for microbiome‐targeted strategies to enhance semen quality for artificial insemination success in swine.

## Introduction

Artificial insemination (AI) is the standard breeding practice for swine operations, with greater than 90% of sows in major pork producing countries worldwide bred using fresh extended semen doses (Waberski et al., 2019). Yet the threat of semen bacterial contamination has been a concern since the development of AI (Koppang and Filseth, 1958). Excessive bacterial presence in semen doses reduce fertility and may contribute to antibiotic resistance in semen extenders (Schulze et al., 2015; Kuster and Althouse, 2016; Rodriguez et al., 2017). Both culture-based and 16S rRNA sequencing methodologies have shown that the presence or elevated relative abundances of pathogenic genera such as *Escherichia*, *Clostridium*, and *Prevotella* are associated with increased sperm cell agglutination and reduced sperm progressive motility (Althouse et al., 2000; Úbeda et al., 2013; McAnally et al., 2023).

Bacterial contamination may occur from animal- or non-animal sources at any point during semen collection, handling, or packaging (Rillo et al., 1998; Althouse and Lu, 2005; Kuster and Althouse, 2016). Hygienic management practices and environmental disinfection can minimize contamination from non-animal sources (e.g., physical surfaces, water lines), but animal-source contamination is more difficult to control (Koppang and Filseth, 1958). Indeed, the boar’s prepuce and surrounding areas are the major sources of contamination. Excessive preputial hair harbors manure and other debris (Althouse et al., 2000), and fluids accumulating in the preputial pouch, such as urine or old semen, can introduce harmful bacteria into the semen during ejaculation (Koppang and Filseth, 1958).

Despite the focus on environmental and preputial sources of bacteria, no information is available regarding the presence or composition of bacterial communities within the boar’s upper urogenital tract, such as the gonads, bladder, and accessory sex glands. Bacterial infections in these organs, such as epididymitis, have been linked to specific bacteria genera such as *Escherichia* or *Mycoplasma* (Azenabor et al., 2015). Recently, characterization of the entire reproductive tract of the bull revealed distinct differences in microbial populations between organs (Retherford et al., 2025), suggesting that unexplored microbial communities may similarly exist in males of other species. These communities may contribute to the bacterial composition of semen and thereby influence boar fertility, as is suggested by the limited data available in human males (Alfano et al., 2018; Molina et al., 2021). Beyond basic community characterization, no studies have considered how the microbial composition of reproductive tissues change in response to stimuli; such changes could provide evidence of community viability or physiological relevance. Major hormonal and anatomical changes occur during puberty that are known to impact microbial communities. For example, in the boar’s gut microbiome, an increase in testosterone from 10 to 22 weeks of age (i.e., before and after puberty) was associated with a reduction in the relative abundance of the phyla Bacteroidetes and Proteobacteria and an increase in the relative abundance of Firmicutes and Actinobacteria (Jiang et al., 2023). Similarly, in the gilt, the gastrointestinal microbiome fluctuates greatly from weaning to first estrus (3 to 32 weeks of age) after which it remains relatively stable throughout subsequent physiological states of gestation and lactation (Gaire et al., 2024). Hence, shifts in microbial communities in reproductive tissues before and after puberty might reflect responsiveness to physiological changes, as has been observed in the known bacterial communities of the gastrointestinal tract.

Therefore, the objectives of this study were: 1) To evaluate the bacterial communities across different urogenital tissues in the boar, including the testicle (testis), epididymis, seminal vesicle, prostate, bulbourethral gland, bladder, and preputial diverticulum (prepuce) and 2) To compare communities before and after puberty to determine the impact of different developmental stages on microbial diversity and composition. It was hypothesized that bacterial populations would shift in relative abundance, alpha-diversity, and beta-diversity due to differing urogenital tissue type and in correlation with increased androgen levels following puberty attainment.

## Methods

Approval of all procedures from the Texas A&M University Institutional Animal Care and Use Committee was acquired for animal acquisition (2020-0323; 2023-0044).

### Animal management and sample collection

Crossbred boars of the same genetic background (Landrace x Yorkshire x Duroc) were born at the Animal Science Swine Unit at Texas A&M University and weaned into a comingled pen in a mechanically ventilated nursery barn at approximately 21 days of age where they were provided *ad libitum* access to water and a conventional pig nursery diet (NRC, 2012). At 74 ± 2 days of age, pre-pubertal boars (*n* = 4) were humanely euthanized and dissected. The daily temperature and humidity range at the time of pre-pubertal boar euthanasia was seasonal for Central Texas (temperature 22.7 to 36.1°C; humidity 32% to 87%). The remaining boars (*n* = 6) were moved into a group pen located in an adjoining naturally ventilated finishing barn on the same farm site (75 meters from the nursery) and fed a conventional finishing diet. Boars were then moved at approximately 228 days of age to individual housing in a standalone naturally ventilated boar stud located on the same farm site (380 meters from the finisher) and fed a conventional finishing diet before humane euthanasia and dissection at 276 ± 3 days of age (post-pubertal attainment). The daily temperature and humidity range at the time of post-pubertal boar euthanasia was seasonal for Central Texas (temperature 27.2 to 35.6 °C; humidity 40% to 79%). In all barns, boars were housed on concrete slats without bedding. These animal numbers are similar to previously published reports of the microbiome of reproductive tract in swine (Hickman-Brown et al., 2025).

Following euthanasia, the intact reproductive tracts of both pre- and post-pubertal boars were removed in a disinfected surgery suite and transferred to dissection tables in the adjoining room. Sterile swabs were collected by rotating the swab within a small incision made in each of the following tissues: testicle (testis), epididymis, bladder, seminal vesicle, prostate, bulbourethral gland, and preputial diverticulum (prepuce). In the case of paired organs (testes, seminal vesicles, and bulbourethral glands), sampling was restricted to the righthand tissue of each animal. One swab sample of the prepuce was not collected from a pre-pubertal boar due to a dissection error. Samples were stored at −80°C until microbial analysis. Additional negative controls were taken by exposing sterile swabs to the air above the necropsied animal in the surgery suite and in the tissue collection room. Additionally, blood samples were collected immediately following euthanasia in 10 mL sodium heparin tubes and kept on ice before centrifuging at 1,500 g for 20 min. Plasma was immediately collected and stored at −20°C until further analysis.

### Testosterone and dihydrotestosterone concentrations

Plasma testosterone concentrations were determined by the Texas A&M Veterinary Medical Diagnostic Laboratory using chemiluminescence. Plasma dihydrotestosterone (DHT) concentrations were determined using a commercially available Porcine DHT ELISA kit (MBS739627, MyBioSource, San Diego, CA). This assay was validated through a series of linearity-of-dilution experiments, and the results were in accordance with the expectations based on manufacturer’s recommendations, with the best dilution being 1:1 (98.6% change from expected reading; Supplementary Table S1). A single assay was completed with samples being diluted 1:1 in PBS (pH 7.0) and run in duplicate. The standard curve R^2^ value was 0.9895 and the intra-assay coefficient of variation was 3.88%.

### DNA extraction and sequencing for microbiome analysis

Microbiome analysis was conducted on all swab samples collected, including testis, epididymis, bladder, seminal vesicle, prostate, bulbourethral gland, preputial diverticulum and negative controls. FERA Diagnostics and Biologicals Corp (College Station, TX) performed DNA extraction and 16S rRNA gene amplicon community sequencing on the V4 hypervariable region. All samples were transferred to a 96-well plate before DNA extraction was conducted using a Mag-Bind Universal Pathogen 96 Kit (Omega Bio-Tek, Norcross, GA) per the manufacturer’s instructions. Specific primers 515F (5ʹ- GTGCCAGCMGCCGCGGTAA-3″) and 806R (5ʹ-GGACTACHVGGGTWTCTAAT-3ʹ) were used to amplify the V4 hypervariable region of the 16S bacterial rRNA genome (Caporaso et al., 2012). Electrophoresis was able to confirm amplicon presence using 1.2% (wt/vol) agarose gels stained with 0.5 mg/mL ethidium bromide. Further, DNA was cleansed for next generation sequencing using magnetic beads (Mag-Bind TotalPure NGS, Omega Bio-Tek, Norcross, GA) per the manufacturer’s instructions. To quantify DNA, spectrophotometry was used with the concentration of pure double-stranded DNA set as A260 of 1.0 was 50 μg/mL. All samples were diluted with ultrapure distilled water to an equal concentration to achieve DNA normalization. Pooled libraries were then analyzed before DNA was quantified with Qubit fluorometric quantification. Following the instructions of the MiSeq System Denature and Dilute Libraries Guide, samples were diluted, denatured, and mixed with PhiX Control v3 (Illumina, San Diego, CA). Lastly, the MiSeq Reagent Kit v2 300 cycles (Illumina, San Diego, CA) and the Illumina MiSeq Platform were used for sequencing. Data files have been placed in the Texas A&M University data repository within the Texas Data Repository: https://doi.org/10.18738/T8/T5JSJC. Additionally, the datasets supporting the conclusions of this article are deposited in the NCBI Short Read Archive (BioProject PRJNA1271515).

### Bioinformatic analysis

Sequence reads were processed for taxon analysis, and quality plots were assessed utilizing the qiime2 pipeline (https://qiime2.org/; Bolyen et al., 2019) on the Grace server provided by Texas A&M High Performance Research Computing. The information provided based on the code ran includes: a node in the long queue, up to 10 hours of compute time, and 6 cores and 35 GB of RAM. For more information, please see https://hprc.tamu.edu/kb/User-Guides/Grace/. DADA2 (Callahan et al., 2016) was subsequently used to remove low-quality sequences with a truncation length of 290. The Greengenes 13_8 database with 99% operational taxonomic units (OTUs) pre-trained classifiers for 16S rRNA was used for taxonomic classification (https://data.qiime2.org/2023.9/common/gg-13-8-99-515-806-nb-classifier.qza). Phylogenetic trees were built using FastTree. Both alpha- (i.e., within sample bacterial diversity measurements) and beta- (i.e., between samples bacterial diversity measurements) diversity metrics were computed. Alpha-diversity was assessed using Shannon’s diversity index, Observed OTUs, Chao1, and Simpson’s diversity index and measures for bacterial community richness and/or evenness to generate comparative boxplots. Beta-diversity was analyzed with unweighted and weighted UniFrac distance metrics to generate PCoA plots (Weinroth et al., 2022).

### Statistical analysis

Bacterial abundances were non-normally distributed. Nonparametric ANOVA in SAS 9.4 (SAS Inst. Inc., Cary, NC) was used for all bacterial abundance data with the independent variables of pubertal status or tissue type. Significance was determined by Kruskal-Wallis test for comparisons between tissue type (>2 categories) and with the pairwise analysis being conducted using the Dwass-Steel-Critchlow-Fligner (DSCF) test for pre-pubertal boars and post-pubertal boars. Significance was determined by Wilcoxon exact test for comparisons between pubertal status (two categories) for each tissue. For alpha diversity, variances were not homogeneous, so the Kruskal-Wallis test was used to confirm the significance of the results using R package ‘ggpubr’. Permutational multivariate ANOVA (PERMANOVA), including unweighted and weighted UniFrac metrics, was used to test the significance for beta diversity using the R function ‘adonis’ in R package ‘vegan’ (Anderson, 2017). Concentrations of testosterone and DHT were analyzed by PROC GLM in SAS 9.4 with the fixed effect of pubertal status and results were reported as least square means ± standard error of the mean (SEM). For correlations using nonparametric data (bacterial abundances and androgen concentrations), Spearman’s correlations were analyzed using PROC CORR in SAS 9.4. Significance was defined as *P *≤ 0.05, and tendencies at 0.05 < *P *< 0.10.

## Results

### 16S rRNA gene amplicon community sequencing summary

After processing and quality filtering, the average number of sequences per sample were 47195 ± 7059 (SEM) and 19844 ± 2204 in pre-pubertal and post-pubertal boar samples, respectively. By tissue type, the average number of sequences per sample were 31102 ± 8960, 26171 ± 10706, 39039 ± 8015, 25343 ± 7209, 27898 ± 6351, 29211 ± 15227, 37529 ± 4167 for the testis, epididymis, bladder, seminal vesicle, prostate, bulbourethral gland, and prepuce samples, respectively. Additionally, the reads from the negative control samples of the necropsy and sampling rooms were 1032 and 685, respectively.

### Bacterial taxonomy—phyla relative abundance

Four phyla exhibited a relative abundance greater than 1% in both pre-pubertal and post-pubertal boars, including Firmicutes, Bacteroidetes, Proteobacteria, and Actinobacteria (Table 1). In pre-pubertal boars, the percent relative abundance of the phylum Firmicutes was greater in the prepuce than the seminal vesicles, prostate, bulbourethral gland, and bladder (*P < *0.05). Additionally, the relative abundance of Firmicutes was greater in the testis and epididymis compared to the prostate and seminal vesicles (*P < *0.05). Alternatively, the relative abundance of the phylum Bacteroidetes was reduced in the prepuce compared to the epididymis, seminal vesicles, prostate, bulbourethral gland, and bladder (*P < *0.01). The relative abundance of Bacteroidetes was also decreased in the testis relative to the seminal vesicles, prostate, bulbourethral gland, and bladder, and was also reduced in the epididymis compared to the seminal vesicles (*P < *0.01). In post-pubertal boars, there were no significant differences between tissues for Firmicutes, Bacteroidetes, or Actinobacteria (*P *> 0.05); however, the bladder had a decreased relative abundance of Proteobacteria compared to the testis, epididymis, seminal vesicle, bulbourethral gland, and prepuce (*P < *0.05).

**Table 1.** Percent relative abundance of phyla (mean ± standard deviation [SD]) between tissue types in pre- and post-pubertal boars

Puberty status^1^	Phylum	Testis	Epididymis	Seminal vesicle	Prostate	Bulbourethral gland	Bladder	Prepuce	*P-*value
Pre-pubertal	Firmicutes	71.2 ± 8.0^ac^	68.5 ± 6.5^ac^	58.4 ± 6.6^b^	59.3 ± 7.0^b^	62.3 ± 4.6^ab^	61.8 ± 3.4^ab^	75.1 ± 9.8^c^	0.0403
	Bacteroidetes	14.2 ± 2.1^ad^	19.9 ± 3.5^ab^	30.1 ± 7.4^c^	27.0 ± 4.3^bc^	23.6 ± 6.3^bc^	25.2 ± 6.6^bc^	9.0 ± 4.6^d^	0.0056
	Proteobacteria	5.6 ± 2.4	5.5 ± 2.3	5.8 ± 2.1	6.5 ± 3.1	6.3 ± 2.6	6.3 ± 2.4	13.5 ± 5.6	0.3551
	Actinobacteria	2.9 ± 2.1	1.6 ± 1.0	1.2 ± 0.7	2.0 ± 1.3	2.8 ± 2.1	1.6 ± 1.1	1.2 ± 0.7	0.7445
Post-pubertal	Firmicutes	42.4 ± 13.7	32.8 ± 13.1	36.9 ± 8.7	44.5 ± 14.5	41.7 ± 6.4	51.5 ± 15.1	44.9 ± 11.6	0.2728
	Bacteroidetes	12.2 ± 6.3	8.8 ± 4.3	21.1 ± 16.6	9.5 ± 8.2	10.8 ± 3.3	9.2 ± 7.2	18.5 ± 9.6	0.1180
	Proteobacteria	26.9 ± 13.7^a^	34.6 ± 18.6^a^	27.5 ± 15.7^a^	21.6 ± 19.8^ab^	32.1 ± 5.3^a^	9.9 ± 4.6^b^	21.7 ± 4.2^a^	0.0164
	Actinobacteria	10.1 ± 8.7	16.3 ± 7.3	9.0 ± 7.0	21.4 ± 19.3	9.4 ± 6.4	26.0 ± 16.0	14.3 ± 7.0	0.2993

1 Sample collection from pre-pubertal boars (*n* = 4) and post-pubertal boars (*n* = 6)

abcd Differences in superscript within row indicate *P*-values < 0.05

Comparisons of bacterial populations by pubertal status within tissue are provided in Table 2. The phyla Firmicutes was greater, and the phyla Proteobacteria was reduced, in pre-pubertal boars compared to post-pubertal boars in the testis, epididymis, seminal vesicles, bulbourethral gland, and prepuce (*P *< 0.05). The phyla Bacteroidetes was present in greater relative abundance in pre-pubertal boars in the epididymis, prostate, bulbourethral gland, and bladder (*P *< 0.05). Finally, the relative abundance of Actinobacteria was greater in the epididymis, prostate, and bladder of post-pubertal boars (*P *< 0.05).

**Table 2.** Percent relative abundance of phyla (mean ± SD) between pre- and post-pubertal boars by tissue type

Tissue	Phylum	Pre-pubertal	Post-pubertal	*P*-value
Testis	Firmicutes	71.2 ± 8.0	42.4 ± 13.7	0.0057
	Bacteroidetes	14.2 ± 2.1	12.2 ± 6.3	0.5747
	Proteobacteria	5.6 ± 2.4	26.9 ± 13.7	0.0116
	Actinobacteria	2.9 ± 2.1	10.1 ± 8.7	0.1665
Epididymis	Firmicutes	68.5 ± 6.5	32.8 ± 13.1	0.0011
	Bacteroidetes	19.9 ± 3.5	8.8 ± 4.3	0.0500
	Proteobacteria	5.5 ± 5.8	34.6 ± 18.6	0.0156
	Actinobacteria	1.6 ± 1.0	16.3 ± 7.3	0.0997
Seminal vesicle	Firmicutes	58.4 ± 6.6	36.9 ± 8.7	0.0032
	Bacteroidetes	30.1 ± 7.4	21.1 ± 16.6	0.3423
	Proteobacteria	5.8 ± 2.1	27.5 ± 15.7	0.0190
	Actinobacteria	1.2 ± 0.7	9.0 ± 7.0	0.0395
Prostate	Firmicutes	59.3 ± 7.0	44.5 ± 14.5	0.0995
	Bacteroidetes	27.0 ± 4.3	9.5 ± 8.2	0.0046
	Proteobacteria	6.5 ± 3.1	21.6 ± 19.8	0.1229
	Actinobacteria	2.0 ± 1.3	21.4 ± 19.3	0.0572
Bulbourethral gland	Firmicutes	62.3 ± 4.6	41.7 ± 6.4	0.0006
	Bacteroidetes	23.6 ± 5.8	10.8 ± 3.3	0.0028
	Proteobacteria	6.3 ± 2.4	32.1 ± 5.3	<0.0001
	Actinobacteria	2.8 ± 1.4	9.4 ± 6.4	0.1079
Bladder	Firmicutes	61.8 ± 3.4	51.5 ± 15.1	0.1599
	Bacteroidetes	25.2 ± 6.6	9.2 ± 7.2	0.0075
	Proteobacteria	6.3 ± 2.4	9.9 ± 4.6	0.2169
	Actinobacteria	1.6 ± 1.1	26.0 ± 16.0	0.0135
Prepuce	Firmicutes	75.1 ± 9.8	44.9 ± 11.6	0.0094
	Bacteroidetes	9.0 ± 4.6	18.5 ± 9.6	0.1689
	Proteobacteria	13.5 ± 5.6	21.7 ± 4.2	0.0547
	Actinobacteria	1.2 ± 0.7	14.3 ± 7.0	0.0123

### Bacterial taxonomy—genera relative abundance

A total of 18 genera exhibited a relative abundance greater than 1% in the pre- and post-pubertal boars (Tables 3 and 4). In pre-pubertal boars, the relative abundances of *Bacteroides*, *Prevotella*, and *Facklamia* differed between tissues (*P < *0.10).

**Table 3.** Percent relative abundance of genera (mean ± SD) between tissue types in pre- and post-pubertal boars

Puberty Status^1^	Genus	Testis	Epididymis	Seminal Vesicle	Prostate	Bulbourethral Gland	Bladder	Prepuce	*P-*value
Pre-pubertal	*Prevotella*	7.5 ± 3.3	10.4 ± 6.3	19.3 ± 10.6	16.4 ± 8.4	14.3 ± 9.1	14.5 ± 10.7	0.2 ± 0.06	0.0889
	*Bacteroides*	2.9 ± 1.2^a^	3.4 ± 1.4^ab^	6.5 ± 2.7^b^	5.4 ± 1.6^b^	4.7 ± 0.8^ab^	4.3 ± 0.5^ab^	0.2 ± 0.09^c^	0.0103
	*Facklamia*	0.2 ± 0.2	0.04 ± 0.03	0.03 ± 0.02	0.07 ± 0.03	0.03 ± 0.01	0.07 ± 0.05	23.3 ± 11.9	0.0788
Post-pubertal	*Streptococcus*	4.1 ± 1.8^ac^	2.9 ± 1.4^a^	2.0 ± 1.7^a^	9.7 ± 4.0^bc^	4.4 ± 2.2^ac^	10.5 ± 6.5^b^	0.8 ± 0.7^a^	0.0077
	*Lactobacillus*	4.2 ± 3.9^xy^	3.8 ± 2.1^xy^	1.3 ± 0.8^xy^	3.1 ± 2.0^y^	2.6 ± 2.5^xy^	4.5 ± 2.8^y^	0.3 ± 0.2^x^	0.0290
	*Escherichia*	3.5 ± 2.6^ac^	11.7 ± 6.0^c^	2.4 ± 1.4^ac^	7.0 ± 5.3^ac^	5.1 ± 1.6^c^	0.5 ± 0.4^a^	0.01 ± 0.01^b^	0.0003
	*Clostridium*	3.1 ± 1.5^xy^	1.7 ± 0.9^xy^	1.7 ± 0.7^xy^	3.4 ± 1.7^xy^	4.7 ± 2.4^xy^	5.8 ± 2.7^x^	0.9 ± 0.7^y^	0.0212
	*Porphyromonas*	0.3 ± 0.2^x^	1.0 ± 0.7^x^	9.3 ± 8.3^xy^	1.0 ± 0.9^x^	2.9 ± 1.6^x^	3.4 ± 2.2^xy^	14.4 ± 5.4^y^	0.0115
	*Blautia*	2.3 ± 2.0	1.6 ± 0.7	3.4 ± 3.2	2.6 ± 1.2	2.9 ± 1.7	2.0 ± 1.7	0.1 ± 0.1	0.0451
	*Turicibacter*	1.0 ± 0.7^xy^	1.7 ± 1.1^xy^	0.3 ± 0.3^x^	2.7 ± 1.9^xy^	0.3 ± 0.3^x^	2.9 ± 1.5^y^	0.1 ± 0.1^x^	0.0045
	*Facklamia*	0.4 ± 0.3^xy^	0.2 ± 0.1^x^	2.2 ± 1.6^xy^	1.3 ± 1.0^xy^	1.4 ± 1.1^xy^	2.4 ± 2.4^y^	9.2 ± 4.5^y^	0.0027
	*Geobacillus*	3.0 ± 2.4^xy^	4.8 ± 2.9^x^	2.3 ± 2.0^xy^	2.2 ± 2.1^xy^	7.3 ± 4.0^x^	0.5 ± 0.3^y^	0.3 ± 0.2^y^	0.0021
	*Caldicellulosiruptor*	7.2 ± 6.3^a^	4.1 ± 4.0^ab^	3.1 ± 1.4^b^	1.5 ± 1.0^b^	2.3 ± 1.3^b^	0.6 ± 0.5^b^	0.02 ± 0.01^b^	0.0027
	*Peptoniphilus*	0.3 ± 0.2^x^	0.4 ± 0.3^x^	4.2 ± 3.1^xy^	0.7 ± 0.6^x^	0.9 ± 0.8^xy^	2.3 ± 1.0^xy^	10.3 ± 6.3^y^	0.0037
	*Corynebacterium*	4.2 ± 2.0^ac^	3.4 ± 1.9^ac^	2.2 ± 1.6^a^	9.0 ± 6.1^bc^	2.4 ± 1.2^ac^	14.7 ± 10.6^b^	3.9 ± 2.4^a^	0.0153
	*Campylobacter*	0.2 ± 0.2^a^	0.01 ± 0.01^a^	5.4 ± 2.9^b^	0.5 ± 0.2^ab^	2.9 ± 2.7^ab^	2.1 ± 1.5^ab^	7.6 ± 3.8^b^	0.0030

1 Sample collection from pre-pubertal boars (*n* = 4) and post-pubertal boars (*n* = 6)

abc Differences in superscript within row indicate *P*-values < 0.05

xy Differences in superscript within row indicate *P*-values 0.05 < *P *≤ 0.10

**Table 4.** Percent relative abundance of genera (mean ± SD) between pre- and post-pubertal boars by tissue type

Tissue	Genus	Pre-pubertal	Post-pubertal	*P*-value
Testis	*Escherichia*	0.05 ± 0.04	3.5 ± 2.6	0.0237
	*Clostridium*	13.1 ± 7.5	3.1 ± 1.5	0.0219
	*Pseudomonas*	0.01 ± 0.01	6.1 ± 2.8	0.0790
	*Blautia*	8.1 ± 3.0	2.3 ± 2.0	0.0061
	*Ruminococcus*	8.7 ± 1.6	2.3 ± 1.0	0.0016
	*Turicibacter*	5.7 ± 3.1	1.0 ± 0.7	0.0148
	*Geobacillus*	0.07 ± 0.06	3.0 ± 2.4	0.0289
	*Caldicellulosiruptor*	0.09 ± 0.08	7.2 ± 6.3	0.0400
Epididymis	*Clostridium*	11.6 ± 8.1	1.7 ± 0.9	0.0891
	*Pseudomonas*	0.02 ± 0.02	5.0 ± 4.1	0.0315
	*Prevotella*	10.4 ± 6.3	0.5 ± 0.4	0.0505
	*Blautia*	8.1 ± 3.4	1.6 ± 1.7	0.0031
	*Ruminococcus*	7.8 ± 1.6	0.5 ± 0.3	<0.0001
	*Bacteroides*	3.4 ± 1.4	1.5 ± 0.6	0.0181
	*Turicibacter*	6.4 ± 4.2	1.7 ± 1.1	0.0677
	*Geobacillus*	0.04 ± 0.03	4.8 ± 2.9	0.0106
	*Caldicellulosiruptor*	0.1 ± 0.1	4.1 ± 3.9	0.0553
	*Corynebacterium*	0.1 ± 0.1	3.4 ± 1.9	0.0081
Seminal Vesicle	*Clostridium*	11.6 ± 6.5	1.7 ± 0.8	0.0517
	*Porphyromonas*	0.3 ± 0.1	9.3 ± 8.3	0.0453
	*Prevotella*	19.3 ± 10.6	3.9 ± 1.8	0.0118
	*Ruminococcus*	8.1 ± 3.3	1.5 ± 1.3	0.0143
	*Bacteroides*	6.5 ± 2.7	2.5 ± 1.3	0.0682
	*Turicibacter*	4.4 ± 3.5	0.3 ± 0.3	0.0990
	*Geobacillus*	0.03 ± 0.02	2.3 ± 2.0	0.0431
	*Caldicellulosiruptor*	0.04 ± 0.02	3.1 ± 1.4	0.0824
	*Peptoniphilus*	0.2 ± 0.1	4.2 ± 3.1	0.0259
	*Corynebacterium*	0.05 ± 0.02	2.2 ± 1.6	0.0196
Prostate	*Clostridium*	10.5 ± 5.5	3.4 ± 1.7	0.0753
	*Pseudomonas*	0.01 ± 0.01	4.2 ± 2.1	0.0983
	*Prevotella*	16.4 ± 8.4	1.8 ± 1.4	0.0045
	*Blautia*	7.4 ± 1.5	2.6 ± 1.2	0.0186
	*Ruminococcus*	8.5 ± 3.4	1.6 ± 0.8	0.0037
	*Bacteroides*	5.4 ± 1.6	1.4 ± 1.3	0.0020
	*Geobacillus*	0.05 ± 0.04	2.2 ± 2.1	0.0501
	*Corynebacterium*	0.08 ± 0.05	9.0 ± 6.1	0.0162
Bulbourethral Gland	*Escherichia*	0.04 ± 0.04	5.1 ± 1.6	0.0005
	*Pseudomonas*	0.01 ± 0.01	5.7 ± 3.5	0.0103
	*Prevotella*	14.3 ± 9.1	1.8 ± 0.8	0.0683
	*Blautia*	7.6 ± 2.2	2.9 ± 1.7	0.0745
	*Ruminococcus*	8.4 ± 3.2	1.5 ± 1.1	0.0068
	*Bacteroides*	4.7 ± 0.8	1.5 ± 1.4	0.0035
	*Turicibacter*	4.6 ± 3.1	0.3 ± 0.3	0.0684
	*Caldicellulosiruptor*	0.15 ± 0.08	2.3 ± 1.3	0.0101
Bladder	*Streptococcus*	2.2 ± 1.3	10.5 ± 6.5	0.0245
	*Lactobacillus*	1.2 ± 0.8	4.5 ± 2.8	0.0490
	*Clostridium*	11.1 ± 5.4	5.8 ± 2.7	0.0716
	*Blautia*	8.3 ± 3.0	2.0 ± 1.7	0.0026
	*Ruminococcus*	8.2 ± 3.1	1.4 ± 1.3	0.0012
	*Bacteroides*	4.3 ± 0.5	1.1 ± 0.9	0.0002
	*Facklamia*	0.1 ± 0.1	2.4 ± 2.3	0.0632
	*Corynebacterium*	0.1 ± 0.02	14.7 ± 10.6	0.0193
Prepuce	*Facklamia*	23.3 ± 11.9	9.2 ± 4.5	0.0480
	*Corynebacterium*	0.04 ± 0.02	3.9 ± 2.4	0.0249

When focusing on the post-pubertal boars, both *Streptococcus* and *Corynebacterium* were observed in greater relative abundance in the bladder than the bulbourethral gland, epididymis, prepuce, seminal vesicle, and testis (*P < *0.05). *Streptococcus* also differed between the prostate and the epididymis, prepuce, and seminal vesicle (*P < *0.05). *Corynebacterium* was decreased in the seminal vesicle compared to the prostate (*P < *0.05). The prepuce’s relative abundance of *Escherichia* was decreased compared to all other tissues as well as decreased in the bladder compared to the epididymis and bulbourethral gland (*P < *0.05). The seminal vesicle and prepuce had increased relative abundances of *Campylobacter* compared to the epididymis and testis (*P < *0.05). The testis had an increased relative abundance of *Caldicellulosiruptor* compared to the bladder, bulbourethral gland, prepuce, prostate, and seminal vesicle (*P < *0.05). There were also differences observed between tissues for *Lactobacillus, Clostridium, Porphyromonas, Turicibacter, Facklamia, Geobacillus*, and *Peptoniphilus.*

Between pre-pubertal and post-pubertal boars, the relative abundances of *Clostridium, Prevotella, Blautia, Ruminococcus, Bacteroides*, and *Turicibacter* were greater in a variety of pre-pubertal tissues compared to post-pubertal tissues (*P *< 0.10). In contrast, the relative abundances of *Escherichia, Porphyromonas, Pseudomonas, Geobacillus, Caldicellulosiruptor*, and *Corynebacterium* were greater in a variety of post-pubertal tissues compared to pre-pubertal tissues (*P *< 0.10). Two tissues exhibited differences in bacterial relative abundance by pubertal status; specifically, the relative abundances of *Streptococcus* and *Lactobacillus* were greater in the bladder of post-pubertal boars than pre-pubertal boars (*P *< 0.05). In the prepuce, the relative abundance of *Facklamia* was greater in pre-pubertal boars compared to post-pubertal boars (*P *< 0.05).

### Alpha- and beta-diversity plots

**Figure 1.**
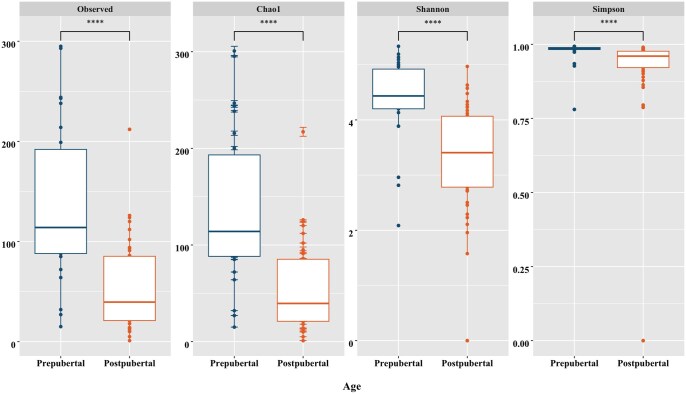
Alpha-diversity metrics (observed OTUs, Chao1, Shannon’s diversity index, Simpson’s diversity index) for differences by pubertal status (**** indicates significance of 0.0001).

**Figure 2.**
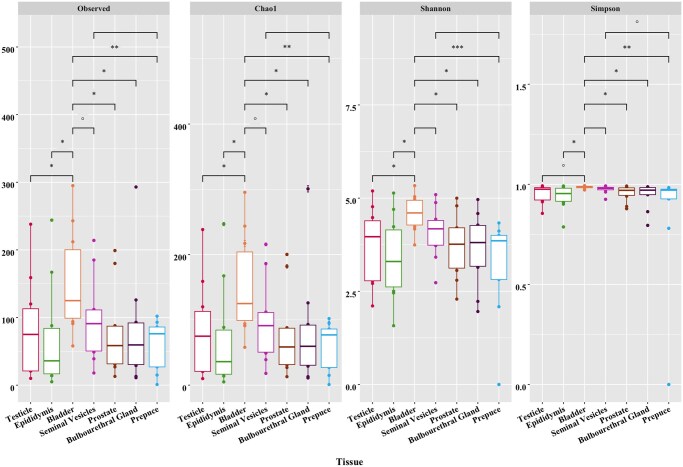
Alpha-diversity metrics (observed OTUs, Chao1, Shannon’s diversity index, Simpson’s diversity index) for differences by tissue type (*** indicates significance of 0.001, ** indicates significance of 0.01, * indicates significance of 0.05, ° indicates a tendency).

All alpha-diversity metrics, including Observed OTUs, Chao1, Shannon’s, and Simpson’s indexes were lower in post-pubertal boars compared to pre-pubertal boars (*P *< 0.01; Fig. 1). By tissue type, all alpha-diversity metrics including Observed OTUs, Chao1, Shannon’s, and Simpson’s indexes were greater in the bladder compared to the testis (*P *< 0.10), epididymis, prostate, bulbourethral gland, and prepuce (*P *< 0.05; Fig. 2). There were tendencies for differences for Observed OTUs and Chao1 between the bladder and seminal vesicles (*P *< 0.10), but no differences were noted for Shannon’s and Simpson’s (*P *> 0.10). In pre-pubertal boars, all metrics including Observed OTUs, Chao1, Shannon’s, and Simpson’s tended to be lower in the prepuce compared to all other tissues (*P *< 0.10; Supplementary Figure S1). In post-pubertal boars, all metrics including Observed OTUs, Chao1, Shannon’s, and Simpson’s were greater in the bladder and prepuce compared to the other tissues (*P *< 0.10; Supplementary Figure S2).

**Figure 3.**
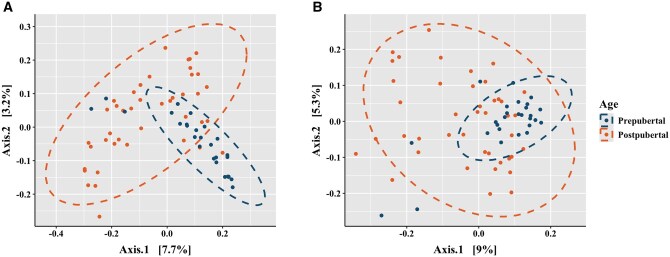
Beta-diversity by unweighted (A) and weighted (B) unique fractions (UniFrac) distance matrices by pubertal status.

**Figure 4.**
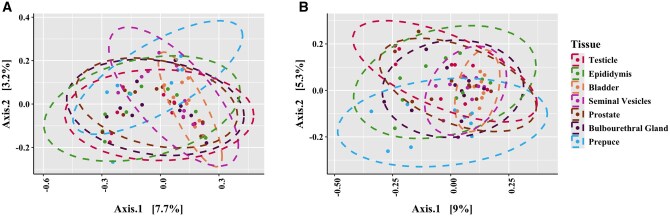
Beta-diversity by unweighted (A) and weighted (B) unique fractions (UniFrac) distance matrices by tissue type.

Distinct clustering was observed by pubertal status using PERMANOVA for both unweighted (*P *< 0.05; Fig. 3A) and weighted (*P *< 0.05; Fig. 3B) UniFrac metrics. Additionally, distinct clustering was observed by tissue type using PERMANOVA for both unweighted (*P *< 0.05; Fig. 4A) and weighted (*P *< 0.05; Fig. 4B) UniFrac metrics. In pre-pubertal boars, there were differences in beta-diversity for unweighted (*P *< 0.05; Supplementary Figure S3A) and weighted (*P *< 0.05; Supplementary Figure S3B) UniFrac metrics. In post-pubertal boars, there were differences in beta-diversity for unweighted (*P *< 0.05; Supplementary Figure S4A) and weighted (*P *< 0.05; Supplementary Figure S4B) UniFrac metrics.

### Testosterone and DHT concentrations and correlations

Testosterone concentrations differed between pre- and post-pubertal boars (0.96 ± 0.36 vs. 3.01 ± 0.26 ng/mL, respectively; *P *< 0.01); however, there were no differences observed in DHT concentrations (9.35 ± 0.52 vs. 8.92 ± 0.42 ng/mL, respectively; *P *= 0.53). Testosterone was correlated with several phyla across the tissues of the reproductive tract. Significant correlations between testosterone and phyla were observed in the testis (Firmicutes [*r* = −0.82; *P *< 0.01] and Proteobacteria [*r* = 0.78; *P *= 0.01]), the epididymis (Firmicutes [*r* = −0.80; *P *= 0.01], Proteobacteria [*r* = 0.95; *P *< 0.01], Bacteroidetes [*r* = −0.85; *P *< 0.01], and Actinobacteria [*r* = 0.63; *P *= 0.07]), the seminal vesicle (Firmicutes [*r* = −0.75; *P *= 0.02], Proteobacteria [*r* = 0.73; *P *= 0.02], and Actinobacteria [*r* = 0.70; *P *= 0.04]), the prostate (Bacteroidetes [*r* = −0.77; *P *= 0.02] and Actinobacteria [*r* = 0.77; *P *= 0.02]), the bulbourethral gland (Proteobacteria [*r* = 0.67; *P *= 0.05] and Bacteroidetes [*r* = −0.78; *P *= 0.01]), the bladder (Bacteroidetes [*r* = −0.85; *P *< 0.01]), and the prepuce (Firmicutes [*r* = −0.76; *P *= 0.03] and Actinobacteria [*r* = 0.83; *P *= 0.01]). No correlations between phyla or genera relative abundances and DHT were found in any tissue (*P *> 0.10). Significant correlations between genera relative abundances and testosterone in all tissues are presented in Supplementary Table S2.

## Discussion

Bacterial contamination reduces the fertility of fresh extended boar semen and can compromise the reproductive performance of the millions of sows bred via AI each year. Non-animal sources of bacteria, such as the collecting dummy, can be disinfected before and after use, while animal sources of contamination, such as the hair around the prepuce or the fluids that collect within, are more difficult to control. Beyond external factors that may alter the seminal microbiota, research is lacking regarding the presence or composition of bacterial communities within the boar’s urogenital tract which may also contribute to the bacterial composition of semen. The objectives of this research were to evaluate the presence and composition of a previously unexplored source of bacteria, the boar’s urogenital tract, and to analyze the shifts in these bacterial communities before and after puberty attainment. Significant differences in both relative abundances and diversity metrics were observed across tissue types and between puberty status, and these results may provide evidence of a viable urogenital microbiome in the boar that furthers understanding of the origins of the semen microbiome.

The male reproductive microbiome has been scarcely investigated, and no studies have previously characterized the bacterial communities of the boar’s urogenital tract. Albeit the phyla observed in the current analyses (Firmicutes, Bacteroidetes, Proteobacteria, and Actinobacteria) are the same that have been identified in the reproductive tissues of other species, such as humans (Alfano et al., 2018; Molina et al., 2021). Indeed, Alfano et al. (2018) reported that in clinically healthy men producing normal sperm, Firmicutes is the major phylum of the testicular parenchyma followed in order of decreasing relative abundance by the phyla Proteobacteria, Actinobacteria, and Bacteroidetes. In the present study, Firmicutes was also present in greatest abundance in the testes of post-pubertal boars, followed in decreasing order by Proteobacteria, Bacteroidetes, and Actinobacteria. In addition, Firmicutes was the primary phylum in all urogenital tissues in the pre-pubertal boar and all tissues except the epididymis of the post-pubertal boar. To our knowledge, no studies in any species have yet compared the relative abundance of bacterial communities of reproductive organs across the entire urogenital tract. This new field of study deserves continued investigation beyond the novel findings of this limited dataset. However, the results of Retherford et al. (2025) demonstrate changes in the diversity and evenness of the bacterial communities in the urethra at different places in the urogenital tract of the bull (i.e., pelvic vs. penile urethra) and suggest that even the reproductive organs including the testes and epididymis contain unique microbial features. These results in the bull support the findings in the present study of unique microbial communities in the urogenital tract in the boar.

The prepuce can harbor a large population of bacteria and is understood to be a common source of contamination during semen collection (Koppang and Filseth, 1958; Althouse et al., 2000; Althouse and Lu, 2005; Goldberg et al., 2013; Kuster and Althouse, 2016). This knowledge led to the development of bacterial contamination minimization techniques such as evacuating the fluid from the prepuce prior to semen collection (Rillo et al., 1998; Althouse et al., 2000). In both pre- and post-pubertal boars, many bacterial genera relative abundances differed between the prepuce and other tissues collected. Interestingly, many bacterial genera detected by previous studies that investigated the causative agents of semen contamination were not elevated in the prepuce in the current analyses (Rillo et al., 1998; Althouse et al., 2000). For example, Rillo et al. (1998) indicated that *Escherichia coli*, *Pseudomonas*, and *Streptococcus* were the main bacteria shared between boar ejaculates and their prepuce. However, in the current study, the bacteria genera that were present in greater relative abundance in the prepuce were *Facklamia* (both pre- and post-pubertal) and *Porphyromonas*, *Peptoniphilus*, and *Campylobacter* (post-pubertal only). These bacteria in the prepuce are considered pathogenic in humans as their abundance has been linked to abnormal, infertile sperm samples, inflammation, and infections in other tissues present throughout the body (Jarvi et al., 1996; Citron et al., 2012; Hou et al., 2013; Weng et al., 2014; Mändar et al., 2017). Both *Campylobacter* and *Porphyromonas* have been found in the boar semen microbiome and are considered potential pathogens for spermatozoa (Gòdia et al., 2020; Li et al., 2023). The animal’s housing space and environment is a likely source of bacteria in the prepuce. For example, of the genera elevated in the prepuce in the current study, *Facklamia* and *Peptoniphilus* have been detected in both skin and feces, respectively (Johnson et al., 2014; Strube et al., 2018). However, it is important to note that in previous swine studies from our group, external contaminants from either the pen or feces have not impacted the microbiota of studied reproductive tissues (Eldridge et al., 2024; Hickman-Brown et al., 2025). Additionally, inconsistencies between preputial bacteria in this study and previous literature could be due to the differences in boars and bacterial identification techniques. The bulk of the scientific literature on the topic has been assembled utilizing culture-based methodologies of bacteriospermic semen samples from clinically unhealthy boars (Rillo et al., 1998; Althouse et al., 2000), whereas the current study used 16S rRNA gene amplicon community sequencing on clinically healthy animals. While culture-based techniques limit the scope of microorganism identification to only about 2% of bacteria, 16S rRNA gene amplicon community sequencing limits individual bacterial species or strains from being determined and is unable to determine if a microbe is alive or dead (Weinroth et al., 2022; Poole et al., 2023).

Many of the bacteria observed in the urogenital tract of the post-pubertal boars in the current study have previously been detected in either the boar or male semen microbiome. For example, the genus *Corynebacterium*, which showed increases in relative abundance after puberty in the epididymis, seminal vesicle, prostate, and bladder, has been observed in semen in several studies, yet with unclear associations with sperm health (Hou et al., 2013; Weng et al., 2014). Interestingly, another genus found in great relative abundance in the post-pubertal boar tract, *Streptococcus*, has been reported as a potential commensal bacterium in the semen of boars (Li et al., 2023) and human males (Weng et al., 2014). Further, the seminal vesicle had elevated levels of *Porphyromonas, Peptoniphilus*, and *Campylobacter* compared to several other tissues. Interestingly, these same bacteria of *Porphyromonas, Peptoniphilus*, and *Campylobacter* were also found to be in greater relative abundance in the prepuce, suggesting that the fluid accumulating in the preputial pouch is likely old seminal fluid. It is important to note that semen was not collected from post-pubertal boars, and the missed opportunity to compare the bacterial communities of semen and the reproductive tract is an acknowledged limitation of the study. Additionally, the difference in bacterial communities between the accessory sex glands and other tissues could be explained by each reproductive organ’s specialized function (McKenzie et al., 1938). Each accessory sex gland provides specific nutrients to an ejaculate, which may attract certain bacterial genera. Moreover, only one bacterial genus, *Caldicellulosiruptor*, was present in the testes at a greater relative abundance than all other tissues collected. This genus, once thought to be related at the class-level to Clostridia, has been previously isolated in the pig gastrointestinal tract (Cheng et al., 2018) but its reproductive function is unknown.

For both alpha- and beta-diversity measurements, differences were observed by pubertal status. Specifically, for alpha diversity, there was lower bacterial diversity in post-pubertal boars compared to pre-pubertal boars. It is important to note that factors such as differing housing and diet requirements for pre- and post-pubertal boars (nursery phase vs. finisher phase) could be associated with the different bacterial communities and diversity of the urogenital tract. Though, one possible explanation could be the difference in testosterone concentrations. In boars, blood testosterone concentration will rise between five to seven months of age, and sexual maturity (ie, puberty) is achieved by six to eight months of age (Allrich et al., 1982; Hill et al., 1998). In the current study, testosterone concentrations had specific correlations with bacterial relative abundance within each reproductive tissue. Specifically, across multiple tissues, testosterone is negatively correlated with the phyla Firmicutes and Bacteroidetes, whereas testosterone was positively correlated with the phyla Proteobacteria and Actinobacteria. In the present study, Firmicutes was the dominant phylum in all urogenital tissues, yet the transition from pre- to post-puberty was marked by a decrease in the relative abundance of Firmicutes, and an increase in the relative abundance of Proteobacteria, in the testes, epididymis, seminal vesicles, and bulbourethral glands. Previous studies have reported Proteobacteria to be the phyla that is present in the greatest relative abundance in both raw (Zhang et al., 2020) and extended (McAnally et al., 2023) boar semen. Specifically, it has been reported that Proteobacteria is in greater relative abundance in extended boar semen with good sperm quality (i.e., sperm progressive motility, sperm morphology) whereas Firmicutes was in greater relative abundance in extended boar semen with poor sperm quality (McAnally et al., 2023). Additionally, the differing correlations between testosterone and bacteria within specific reproductive tissues could be due to different tissues potentially being exposed to varying levels of testosterone or respond differently to testosterone. For example, it has been previously shown in pre-pubertal castrated boars that subcutaneous injections of testosterone supported the growth and secretory activity of the prostate but not of the seminal vesicles or bulbourethral gland (Booth, 1980). Moreover, these associations between testosterone and bacterial relative abundance may be due, in part, to the fact that steroid hormones can influence toll-like receptor (TLR) expression in the body (Norata et al., 2006; Rettew et al., 2008). For example, *in vitro* studies have observed that testosterone reduces TLR4 in macrophages, and in *in vivo* studies there is an increase in TLR4 when endogenous testosterone is removed (Quintar et al., 2006; Rettew et al., 2008). Toll-like receptor 4 predominantly recognizes lipopolysaccharide, which is characteristic of gram-negative bacteria (e.g., *Escherichia* and *Pseudomonas*; Hoshino et al., 1999; Janeway and Medzhitov, 2002). Interestingly, in fecal samples collected from Meishan boars (10 and 22 weeks of age), testosterone was positively associated with Firmicutes and negatively associated with Proteobacteria (Jiang et al., 2023). These discrepancies in findings to the current study could be due to differences in sample type (reproductive tissues vs. fecal), breed (Landrace × Yorkshire × Duroc vs. Meishan), and/or boar age (2 months [8 weeks] and 8 months [56 weeks] vs. 10 to 22 weeks of age).

Though beyond the scope of the current study, an important discussion point is where these bacterial communities originate and how do they spread and establish in the urogenital tract. A working hypothesis is viable bacteria migrate from the gastrointestinal tract via hematogenous spread otherwise known as bacterial translocation (Berg, 1999). Potentially, this process occurs within the first 24 to 36 hours of life before gut closure in which large molecules can transfer across the intestinal wall (Everaert et al., 2017; Weström et al., 2020). These microbes are then trafficked through hematogenous spread from the neonate’s gut to the urogenital environment and are defined as the pioneer microbiome of the urogenital tract (i.e., the initial colonization of microbial organisms; Eldridge et al., 2024). These microbes then adapt to the environment and the succession of the bacterial community begins as the animal matures. Similarly, it is important to note that in the current study, the daily temperature and humidity ranges for both pre- and post-pubertal boars were seasonal for Central Texas; however, these boars were exposed to heat stress conditions (Baker, 2004). It has been previously shown that environmental conditions (i.e., seasonal differences in temperature and humidity) can alter bacterial composition of boar semen (Zhang et al., 2020). Therefore, because heat stress conditions can alter the integrity of the intestinal epithelial barrier (Mayorga et al., 2019), this could permit bacterial translocation via the bloodstream to the various reproductive tissues examined in the current study.

## Conclusions

Based on these preliminary results, the boar urogenital tissues have distinct bacterial populations from each other despite their proximity. Potentially, the origin of bacteria in boar semen may originate from several sources, including the environment and/or the host’s own reproductive tract. In addition, bacterial communities and diversity shift within the boar reproductive tract after pubertal attainment, possibly due to increases in testosterone concentrations, though future studies should be conducted to corroborate these findings and control for various environmental conditions (i.e., housing, diet, temperature, etc.). Further, future studies comparing the bacterial compositions of boar reproductive tissues to the animal’s ejaculate could provide greater insight into the origin of bacteria within boar semen.

## Supplementary Material

skaf336_Supplementary_Data

## Abbreviations:

AI: Artificial insemination
DHT: Dihydrotestosterone
DSCF: Dwass-Steel-Critchlow-Fligner
PERMANOVA: Permutational multivariate ANOVA
OTU: Operational taxonomic units
SD: Standard deviation
SEM: Standard error of the mean
TLR: Toll-like receptor
UniFrac: Unique fractions

## References

[skaf336-B1] Alfano M et al 2018. Testicular microbiome in azoospermic men-first evidence of the impact of an altered microenvironment. Hum. Reprod. 33(7):1212–1217. 10.1093/humrep/dey116 29850857 PMC6012977

[skaf336-B2] Allrich RD , Christenson RK, Ford JJ, Zimmerman DR. 1982. Pubertal development of the boar: testosterone, estradiol-17 beta, cortisol and LH concentrations before and after castration at various ages. J. Anim. Sci. 55(5):1139–1146. 10.2527/jas1982.5551139x 7174554

[skaf336-B3] Althouse GC , Kuster CE., Clark SG., Weisiger RM. 2000. Field investigations of bacterial contaminants and their effects on extended porcine semen. Theriogenology. 53(5):1167–1176. 10.1016/S0093-691X(00)00261-2 10798493

[skaf336-B4] Althouse GC , Lu KG. 2005. Bacteriospermia in extended porcine semen. Theriogenology. 63(2):573–584. 10.1016/j.theriogenology.2004.09.031 15626417

[skaf336-B5] Anderson MJ. 2017. Permutational Multivariate Analysis of Variance (PERMANOVA). Wiley Stats Ref: Stat Ref Online. 10.1002/9781118445112.STAT07841

[skaf336-B6] Azenabor A , Ekun AO., Akinloye O. 2015. Impact of inflammation on male reproductive tract. J. Reprod. Infertil. 16(3):123–129. 26913230 PMC4508350

[skaf336-B7] Baker JE. 2004. Effective environmental temperature. JSHAP. 12(3):140–143.

[skaf336-B8] Berg RD. 1999. Bacterial translocation from the gastrointestinal tract. Adv. Exp. Med. Biol. 473:11–30. 10.1007/978-1-4615-4143-1_2 10659341

[skaf336-B9] Bolyen E. et al 2019. Author correction: Reproducible, interactive, scalable and extensible microbiome data science using QIIME 2. Nat. Biotechnol. 37(9):1091. 10.1038/s41587-019-0252-6 31399723

[skaf336-B10] Booth WD. 1980. A study of some major testicular steroids in the pig in relation to their effect on the development of male characteristics in the prepubertally castrated boar. J. Reprod. Fertil. 59(1):155–162. 10.1530/jrf.0.0590155 7401032

[skaf336-B11] Callahan BJ. et al 2016. DADA2: High-resolution sample inference from illumina amplicon data. Nat. Methods. 13(7):581–583. 10.1038/nmeth.3869 27214047 PMC4927377

[skaf336-B12] Caporaso JG. et al 2012. Ultra-high-throughput microbial community analysis on the illumina HiSeq and MiSeq platforms. Isme J. 6(8):1621–1624. 10.1038/ismej.2012.8 22402401 PMC3400413

[skaf336-B13] Cheng P. et al 2018. Exploratory analysis of the microbiological potential for efficient utilization of fiber between lantang and duroc pigs. Front. Microbiol. 9:1342. 10.3389/fmicb.2018.01342 29988353 PMC6023970

[skaf336-B14] Citron DM. , Tyrrell KL., Goldstein EJ. 2012. Peptoniphilus coxii sp. nov. and Peptoniphilus tyrrelliae sp. nov. isolated from human clinical infections. Anaerobe. 18(2):244–248. 10.1016/j.anaerobe.2011.11.008 22178538

[skaf336-B15] Eldridge LK. et al 2024. Maternal versus environmental contributions to the piglet pioneer microbiome. Reprod. Fertil. 5(3):e240009. 10.1530/RAF-24-0009 38847820 PMC11301562

[skaf336-B16] Everaert N. et al 2017. A review on early gut maturation and colonization in pigs, including biological and dietary factors affecting gut homeostasis. Anim. Feed Sci. Technol. 233:89–103. 10.1016/j.anifeedsci.2017.06.011

[skaf336-B17] Gaire TN. et al 2024. Temporal dynamics of the fecal microbiome in female pigs from early life through estrus, parturition, and weaning of the first litter of piglets. Anim. Microbiome. 6(1):7. 10.1186/s42523-024-00294-8 38383422 PMC10882843

[skaf336-B18] Gòdia M. et al 2020. A pilot RNA-seq study in 40 pietrain ejaculates to characterize the porcine sperm microbiome. Theriogenology. 157:525–533. 10.1016/j.theriogenology.2020.08.001 32971422

[skaf336-B19] Goldberg AM. et al 2013. Risk factors for bacterial contamination during boar semen collection. Res. Vet. Sci. 95(2):362–367. 10.1016/j.rvsc.2013.06.022 23891384

[skaf336-B20] Hickman-Brown KJ. et al 2025. Stage of pregnancy impacts the bacterial communities of reproductive and placental tissues in gilts. J. Anim. Sci. 103 10.1093/jas/skaf159 PMC12132795 40336167

[skaf336-B21] Hill G. et al 1998. Tri-state swine nutrition guide. The Ohio State University Bulletin. 869

[skaf336-B22] Hoshino K. et al 1999. Cutting edge: Toll-like receptor 4 (TLR4)-deficient mice are hyporesponsive to lipopolysaccharide: evidence for TLR4 as the lps gene product. J. Immunol. 162(7):3749–3752. 10.4049/jimmunol.162.7.3749 10201887

[skaf336-B23] Hou D. et al 2013. Microbiota of the seminal fluid from healthy and infertile men. Fertil. Steril. 100(5):1261–1269. 10.1016/j.fertnstert.2013.07.1991 23993888 PMC3888793

[skaf336-B24] Janeway CA. Jr , Medzhitov R. 2002. Innate immune recognition. Annu. Rev. Immunol. 20:197–216. 10.1146/annurev.immunol.20.083001.084359 11861602

[skaf336-B25] Jarvi K. et al 1996. Polymerase chain reaction-based detection of bacteria in semen. Fertil. Steril. 66(3):463–467. 8751749

[skaf336-B26] Jiang X. et al 2023. Fecal microbial composition associated with testosterone in the development of meishan male pigs. Front. Microbiol. 14:1257295. 10.3389/fmicb.2023.1257295 38053550 PMC10694212

[skaf336-B27] Johnson CN. , Whitehead TR., Cotta MA., Rhoades RE., Lawson PA. 2014. Peptoniphilus stercorisuis sp. nov., isolated from a swine manure storage tank and description of peptoniphilaceae fam. nov. Int. J. Syst. Evol. Microbiol. 64(Pt 10):3538–3545. 10.1099/ijs.0.058941-0 25056296

[skaf336-B28] Koppang N. , Filseth O. 1958. The bacterial flora of semen and prepuce in boars. Nord. Vet. Med. 10:603–609.

[skaf336-B29] Kuster CE. , Althouse GC. 2016. The impact of bacteriospermia on boar sperm storage and reproductive performance. Theriogenology. 85(1):21–26. 10.1016/j.theriogenology.2015.09.049 26525397

[skaf336-B30] Li D. , Xu Y., Wang M., Fang S., Li SH., Cui Y. 2023. Differences of semen microbiota among breeding boars with different reproductive ages. J. Anim. Sci. 101: skad247. 10.1093/jas/skad247 PMC10424712 37478469

[skaf336-B31] Mändar R. et al 2017. Seminal microbiome in men with and without prostatitis. Int. J. Urol. 24(3):211–216. 10.1111/iju.13286 28147438

[skaf336-B32] Mayorga EJ , Renaudeau D., Ramirez BC., Ross JW., Baumgard LH. 2019. Heat stress adaptations in pigs. Anim Front. 9(1):54–61. 10.1093/af/vfy035 32002240 PMC6951998

[skaf336-B33] McAnally BE. , Smith MS., Wiegert JG., Palanisamy V., Chitlapilly Dass S., Poole RK. 2023. Characterization of boar semen microbiome and association with sperm quality parameters. J. Anim. Sci. 101:skad243. 10.1093/jas/skad243 37464945 PMC10393202

[skaf336-B34] McKenzie FF. , Miller JC., Bauguess LC. 1938. The reproductive organs and semen of the boar. University of Missouri, College of Agriculture, Agricultural Experiment Station.

[skaf336-B35] Molina NM. et al 2021. Assessing the testicular sperm microbiome: a low-biomass site with abundant contamination. Reprod. Biomed. Online. 43(3):523–531. 10.1016/j.rbmo.2021.06.021 34344601

[skaf336-B36] Nitsche-Melkus E. , Bortfeldt R., Jung M., Schulze M. 2020. Impact of hygiene on bacterial contamination in extended boar semen: an eight-year retrospective study of 28 european AI centers. Theriogenology. 146:133–139. 10.1016/j.theriogenology.2019.11.031 31806246

[skaf336-B37] Norata GD. , Tibolla G., Seccomandi PM., Poletti A., Catapano AL. 2006. Dihydrotestosterone decreases tumor necrosis factor-alpha and lipopolysaccharide-induced inflammatory response in human endothelial cells. J. Clin. Endocrinol. Metab. 91(2):546–554. 10.1210/jc.2005-1664 16317058

[skaf336-B38] NRC. 2012. Nutrient requirements of swine. 11th rev. ed. Washington, DC: Natl. Acad. Press. 10.17226/13298

[skaf336-B39] Poole RK. et al 2023. Reproductive microbiomes in domestic livestock: Insights utilizing 16S rRNA gene amplicon community sequencing. Animals (Basel). 13(3):485. 10.3390/ani13030485 36766374 PMC9913168

[skaf336-B40] Quintar AA. , Roth FD., De Paul AL., Aoki A., Maldonado CA. 2006. Toll-like receptor 4 in rat prostate: modulation by testosterone and acute bacterial infection in epithelial and stromal cells. Biol. Reprod. 75(5):664–672. 10.1095/biolreprod.106.053967 16870940

[skaf336-B41] Retherford S. , Woodruff KL., Dittoe DK., Block J. 2025. Characterization of the extent and composition of the bull reproductive microbiome. Reprod. Fertil. Dev. 37 10.1071/RDv37n1Ab158

[skaf336-B42] Rettew JA. , Huet-Hudson YM., Marriott I. 2008. Testosterone reduces macrophage expression in the mouse of toll-like receptor 4, a trigger for inflammation and innate immunity. Biol. Reprod. 78(3):432–437. 10.1095/biolreprod.107.063545 18003947

[skaf336-B43] Rillo MS. , Shokouhi V., Boix EG., Hernandez-Gil R. 1998. Contamination of semen doses and its possible relationship with the bacterial flora of the prepuce. In: 15th International Pig Veterinary Society Congress; p 60.

[skaf336-B44] Rodriguez AL. , Van Soom A., Arsenakis I., Maes D. 2017. Boar management and semen handling factors affect the quality of boar extended semen. Porcine Health Manag. 3:15. 10.1186/s40813-017-0062-5 28770098 PMC5525438

[skaf336-B45] Schulze M. , Ammon C., Rüdiger K., Jung M., Grobbel M. 2015. Analysis of hygienic critical control points in boar semen production. Theriogenology. 83(3):430–437. 10.1016/j.theriogenology.2014.10.004 25459424

[skaf336-B46] Singleton W , Flowers B. 2001. Managing boars in artificial insemination centers. Pork information gateway: Factsheet. Des Moines, IA: National Pork Board.

[skaf336-B47] Strube ML. , Hansen JE., Rasmussen S., Pedersen K. 2018. A detailed investigation of the porcine skin and nose microbiome using universal and Staphylococcus specific primers. Sci. Rep. 8(1):12751. 10.1038/s41598-018-30689-y 30143677 PMC6109091

[skaf336-B48] Úbeda JL. et al 2013. Adverse effects of members of the enterobacteriaceae family on boar sperm quality. Theriogenology. 80(6):565–570. 10.1016/j.theriogenology.2013.05.022 23827823

[skaf336-B49] Waberski D. , Riesenbeck A., Schulze M., Weitze KF., Johnson L. 2019. Application of preserved boar semen for artificial insemination: past, present and future challenges. Theriogenology. 137:2–7. 10.1016/j.theriogenology.2019.05.030 31186127

[skaf336-B50] Weinroth MD. et al 2022. Considerations and best practices in animal science 16S ribosomal RNA gene sequencing microbiome studies. J. Anim. Sci. 100(2):skab346. 10.1093/jas/skab346 35106579 PMC8807179

[skaf336-B51] Weng SL. et al 2014. Bacterial communities in semen from men of infertile couples: metagenomic sequencing reveals relationships of seminal microbiota to semen quality. PLoS One. 9(10):e110152. 10.1371/journal.pone.0110152 25340531 PMC4207690

[skaf336-B52] Weström B. , Arévalo Sureda E., Pierzynowska K., Pierzynowski SG., Pérez-Cano FJ. 2020. The immature gut barrier and its importance in establishing immunity in newborn mammals. Front. Immunol. 11:1153. 10.3389/fimmu.2020.01153 32582216 PMC7296122

[skaf336-B53] Zhang J. et al 2020. Genomic sequencing reveals the diversity of seminal bacteria and relationships to reproductive potential in boar sperm. Front. Microbiol. 11:1873. 10.3389/fmicb.2020.01873 32903829 PMC7438901

