# Supplementary material for: Puberty status influences bacterial communities of the boar urogenital tract
Source: J Anim Sci. 2025 Sep 27;103:skaf336. doi: 10.1093/jas/skaf336 (PMC12569513; doi:10.1093/jas/skaf336)

**Supplementary Table 1.** Porcine DHT ELISA kit (MBS739627, MyBioSource) validation through a series of linearity-of-dilution experiments.

| **Sample** | **Reading (Average OD)** | **Expected Reading** | **% Change** |
| --- | --- | --- | --- |
| Neat (Sample A) | 0.339 | N/A | N/A |
| 1:1 (Sample A) | 0.659 | 0.678 | 97.2% |
| 1:2 (Sample A) | 0.926 | 1.318 | 70.3% |
| 1:4 (Sample A) | 1.091 | 1.852 | 58.9% |
|  |  |  |  |
| Neat (Sample B) | 0.191 | N/A | N/A |
| 1:1 (Sample B) | 0.4105 | 0.382 | 107.5% |
| 1:2 (Sample B) | 0.6905 | 0.821 | 84.1% |
| 1:4 (Sample B) | 1.0495 | 1.381 | 76.0% |
|  |  |  |  |
| Neat (Sample C) | 0.2665 | N/A | N/A |
| 1:1 (Sample C) | 0.5325 | 0.533 | 99.9% |
| 1:2 (Sample C) | 0.905 | 1.065 | 85.0% |
| 1:4 (Sample C) | 1.1095 | 1.81 | 61.3% |

**Supplementary Table 2.** Spearman Correlation Coefficients between genera relative abundances and testosterone for each tissue.

| **Tissue** | **Genus** | **Correlation Coefficient** | ***P*-value** |
| --- | --- | --- | --- |
| Testicle | *Escherichia* | 0.817 | 0.007 |
|  | *Clostridium* | -0.667 | 0.049 |
|  | *Pseudomonas* | 0.917 | 0.001 |
|  | *Prevotella* | -0.731 | 0.025 |
|  | *Blautia* | -0.917 | 0.001 |
|  | *Ruminococcus* | -0.883 | 0.002 |
|  | *Turicibacter* | -0.828 | 0.006 |
|  | *Geobacillus* | 0.883 | 0.002 |
|  | *Caldicellulosiruptor* | 0.883 | 0.002 |
|  |  |  |  |
| Epididymis | *Escherichia* | 0.900 | 0.001 |
|  | *Clostridium* | -0.850 | 0.004 |
|  | *Pseudomonas* | 0.767 | 0.016 |
|  | *Prevotella* | -0.650 | 0.058 |
|  | *Blautia* | -0.933 | 0.0002 |
|  | *Ruminococcus* | -0.915 | 0.001 |
|  | *Bacteroides* | -0.683 | 0.042 |
|  | *Geobacillus* | 0.817 | 0.007 |
|  | *Campylobacter* | -0.895 | 0.001 |
|  |  |  |  |
| Seminal Vesicle | *Escherichia* | 0.617 | 0.077 |
|  | *Clostridium* | -0.883 | 0.002 |
|  | *Pseudomonas* | 0.800 | 0.010 |
|  | *Prevotella* | -0.720 | 0.029 |
|  | *Blautia* | -0.783 | 0.013 |
|  | *Ruminococcus* | -0.633 | 0.067 |
|  | *Bacteroides* | -0.817 | 0.007 |
|  | *Turicibacter* | -0.883 | 0.002 |
|  | *Geobacillus* | 0.600 | 0.088 |
|  | *Caldicellulosiruptor* | 0.783 | 0.013 |
|  |  |  |  |
| Prostate | *Porphyromonas* | -0.600 | 0.088 |
|  | *Pseudomonas* | 0.783 | 0.013 |
|  | *Prevotella* | -0.717 | 0.030 |
|  | *Bacteroides* | -0.917 | 0.001 |
|  | *Facklamia* | 0.833 | 0.005 |
|  | *Geobacillus* | 0.617 | 0.077 |
|  | *Corynebacterium* | 0.767 | 0.016 |
|  | *Campylobacter* | -0.762 | 0.017 |
|  |  |  |  |
| Bulburethral Gland | *Escherichia* | 0.683 | 0.042 |
|  | *Pseudomonas* | 0.883 | 0.002 |
|  | *Prevotella* | -0.750 | 0.020 |
|  | *Ruminococcus* | -0.778 | 0.014 |
|  | *Bacteroides* | -0.717 | 0.030 |
|  | *Turicibacter* | -0.845 | 0.004 |
|  | *Geobacillus* | 0.817 | 0.007 |
|  | *Caldicellulosiruptor* | 0.700 | 0.036 |
|  | *Corynebacterium* | 0.667 | 0.050 |
|  | *Campylobacter* | -0.731 | 0.025 |
|  |  |  |  |
| Bladder | *Streptococcus* | 0.750 | 0.020 |
|  | *Lactobacillus* | 0.717 | 0.030 |
|  | *Prevotella* | -0.700 | 0.036 |
|  | *Blautia* | -0.650 | 0.058 |
|  | *Ruminococcus* | -0.617 | 0.077 |
|  | *Bacteroides* | -0.833 | 0.005 |
|  | *Facklamia* | 0.783 | 0.013 |
|  | *Corynebacterium* | 0.767 | 0.016 |
|  |  |  |  |
| Prepuce | *Escherichia* | 0.655 | 0.078 |
|  | *Pseudomonas* | 0.671 | 0.069 |
|  | *Prevotella* | 0.643 | 0.086 |
|  | *Geobacillus* | -0.857 | 0.007 |
|  | *Corynebacterium* | 0.833 | 0.010 |

**Supplementary Figure 1.** Alpha diversity metrics (observed OTUs, Chao1, Shannon’s diversity index, Simpson’s diversity index) for differences by tissue in pre-pubertal boars (° indicates a tendency).


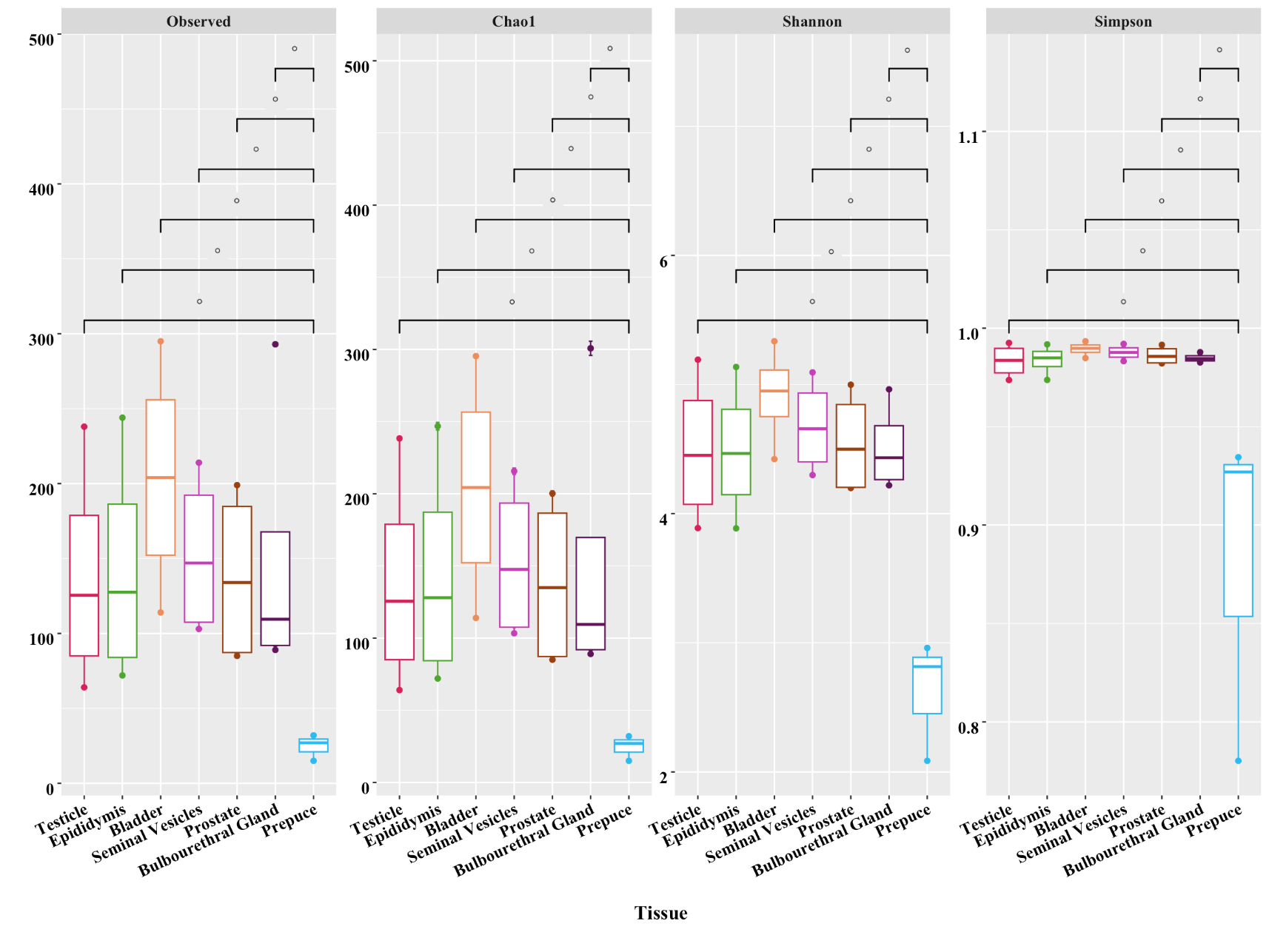


**Supplementary Figure 2.** Alpha diversity metrics (observed OTUs, Chao1, Shannon’s diversity index, Simpson’s diversity index) for differences by tissue in post-pubertal boars (** indicates significance of 0.01, * indicates significance of 0.05, ° indicates a tendency).


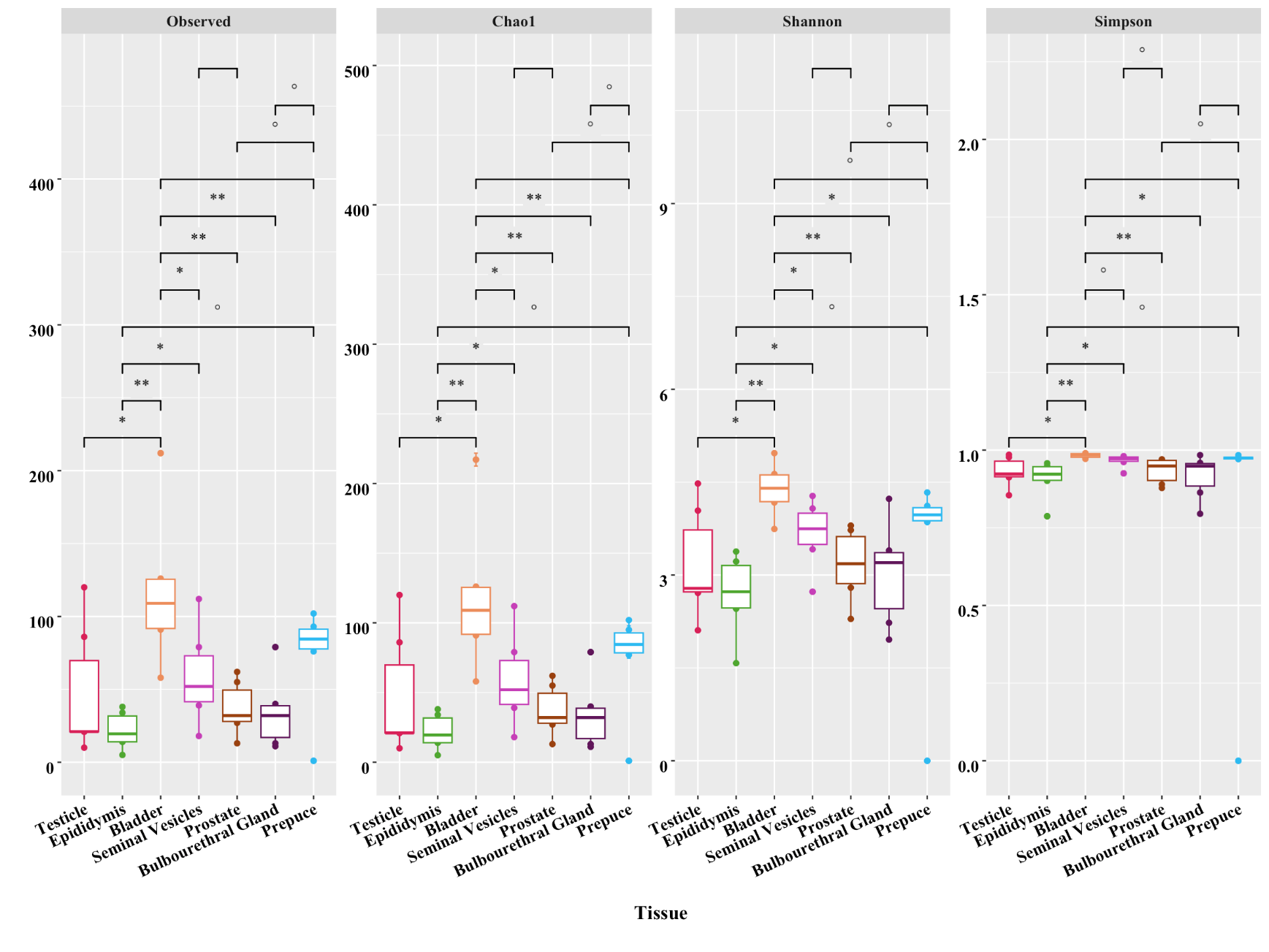


**Supplementary Figure 3.** Beta diversity by unweighted (A) and weighted (B) unique fractions (UniFrac) distance matrices by tissue in pre-pubertal boars.


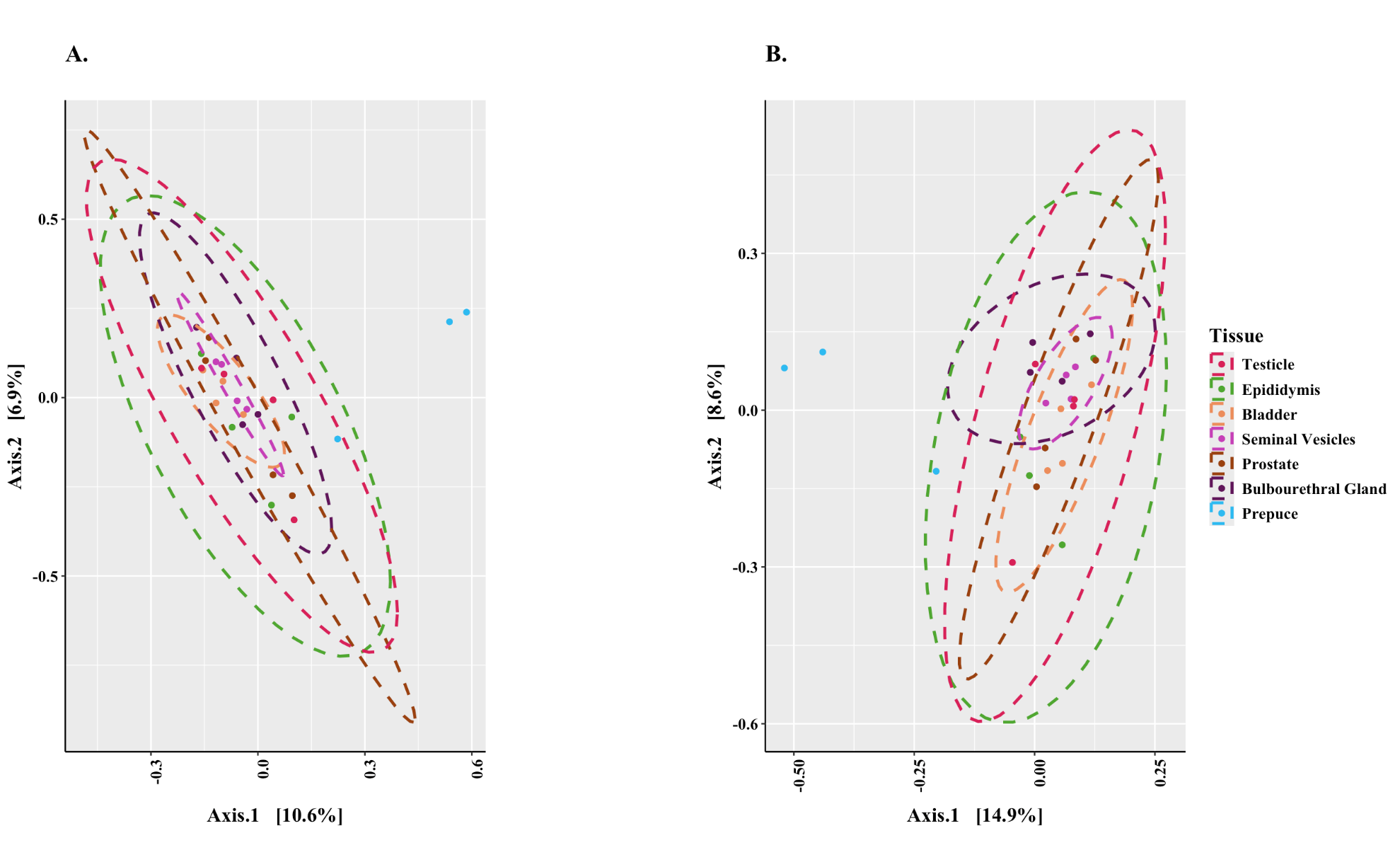


**Supplementary Figure 4.** Beta diversity by unweighted (A) and weighted (B) unique fractions (UniFrac) distance matrices by tissue in post-pubertal boars.


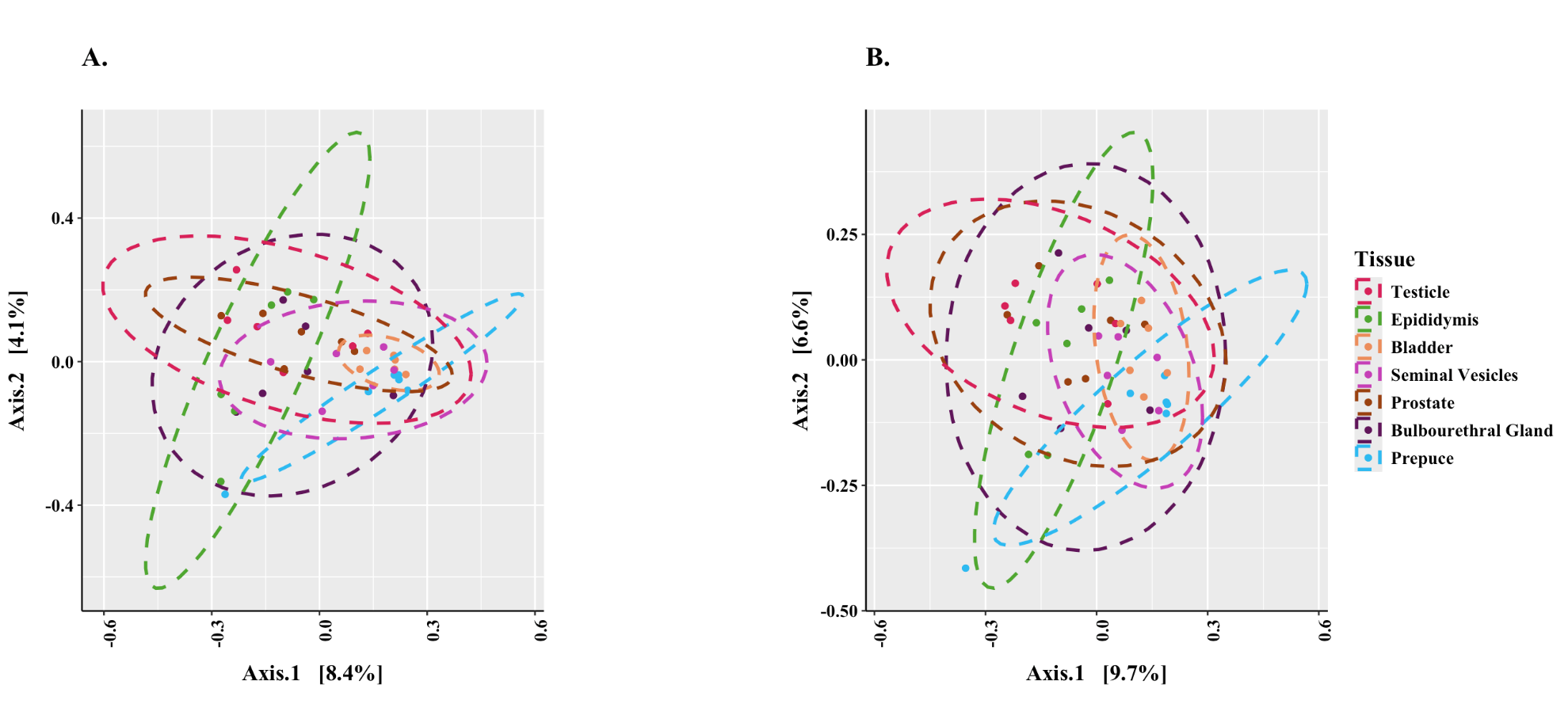

Supplement: skaf336_Supplementary_Data [file skaf336_supplementary_data.docx]
